# Supplementary material for: Polyandry blocks gene drive in a wild house mouse population
Source: Nat Commun. 2020 Nov 4;11:5590. doi: 10.1038/s41467-020-18967-8 (PMC7643059; doi:10.1038/s41467-020-18967-8)
Supplement: Supplementary file 1 — Supplementary Information [file 41467_2020_18967_MOESM1_ESM.pdf]

Supplementary Information for

Polyandry Blocks Gene Drive in a Wild House Mouse Population

Manser et al.

Contents

1 Estimating Heritability of Polyandry 2

2 Estimating Selection on Polyandry 5

3 A Model of Polyandry and Gene Drive 8

A Monandry 9

B Polyandry 10

4 Estimating Model Parameters 12

List of Figures

1 Additive Genetic Variance, Individual Variance, and Heritability of Polyandry 4

2 The Impact of Polyandry on Female Fitness 6

3 Adult Population Size During the Study Period 17

List of Tables

1 Model Selection Table: The Impact of Polyandry on Female Fitness 7

2 Model Parameter Estimation 13

3 Mating Table and Expected Paternity Shares 15

4 Model Selection Table: The Impact of Polyandry on Male Fitness 18

Supplementary Note 1

Estimating Heritability of Polyandry

In analysis 1), we modelled the frequency of polyandry in the population using a generalised linear mixed effects model (GLMM) that uses pairwise relatedness matrix as a random effect (also known as an animal model). In this supplementary section, we provide additional detail on the implementation of the model.

Random Effect Structure Two random effect variables were included in the model, the random additive genetic effect (V_A) and maternal identity (V_{PE}).

1. To estimate the amount of phenotypic trait variation V_P that can be attributed to heritable genetic variation among females (V_A) we used the parentage analysis (see Methods of main text) to construct a pairwise relatedness matrix of all 225 females in the data set. From the entire pedigree, we removed all non-informative animals (animals that did not reproduce and/or were not responsible for a link between two informative animals) using the `prunePed` function in the R package `MCMCglmm`[4]. The remaining pedigree contained a total of 451 individuals, of which 41 individuals (9%) were treated as founders due to unknown maternal and paternal links. The average depth of the pedigree was 8.44, the maximum depth equaled 17. About 50% of the individuals had an inbreeding coefficient greater than 0, and the average degree of inbreeding was 0.065. The mean pairwise relatedness was 0.068, and about 20% of dyads had values greater than 0.125. Pedigree summary statistics were produced with the help of the `Pedantics` package in R[11].

2. Several females in the data set reproduced more than once. We accounted for such repeated female reproduction by fitting female identity as an additional random effect variable. This allowed us to test for potential, systematic differences in polyandry rates among females. In quantitative genetics studies, this variance component is usually termed V_{PE} (for permanent environment).

Fixed Effect Structure Additional to the random effects variables, we fitted a number of additional predictors as fixed explanatory variables. In a full model, we investigated the effect of female t genotype (+/+ and +/t), adult population size (Supplementary Figure 3) and average monthly temperature at the time when the litter was born, as well as the size of the litter (without interactions). Note that the inclusion of litter size as an explanatory variable is of crucial importance

here, as we expect a higher probability of genetic polyandry in larger litters based on chance alone (because of the reduced sampling error in larger litters).

Implementation Details Bayesian analyses require the specification of prior probability distributions for all random and fixed predictors used in the model. We used relatively uninformative priors for both fixed effects (normally distributed with a mean of 0 and a variance of 10^8) and random effects (inverse Wishart distributed, with variances set to 1 and degree of belief of 1). Because estimated variance components V_A and V_E were close to zero, we used parameter expansion to ensure proper mixing of the posterior chains. Model outcomes were robust with regard to the (reasonable) choice of prior distribution. Note that residual variance V_R cannot be estimated in binary models[3;12;13] and were therefore set to a fixed, arbitrary value of 1. It is important to mention that, while the arbitrary choice of V_R does affect absolute estimates of the other variance components V_A and V_{PE}, it has only minor effects on the relative magnitude of the three variance components. Hence, the choice of V_R should not affect our heritability estimates. We run all models for 10^6 iterations, with a burn-in of 5,000 and a thinning interval of 3,000 to avoid autocorrelation among the samples from the posterior distribution. After running a full-model including all fixed and random variables, we removed non-significant fixed factors in a stepwise fashion.

Calculating Heritability Heritability h^2 is defined as the proportion of phenotypic variance V_P that is accounted for by additive genetic variance V_A. We were interested in the heritability of a female's propensity for genetic polyandry. In the model here, the propensity is not estimated on the scale at which the trait was measured (data-scale), but on the underlying logit-scale (latent-scale). Accordingly, we used the following expression to calculate the latent-scale heritability of genetic polyandry

$$h^2 = \frac{V_A}{V_A + V_{PE} + V_R + \frac{\pi^2}{3}} \quad (1)$$

where the logistic variance is proportional to $\frac{\pi^2}{3}$[12].

Limitations There are several potential explanations for the low heritability measured here. One possibility is that there is considerable additive genetic variation V_A, but this variation is negligible in view of the far greater, other sources of variation (i.e. V_R, V_{PE}). Unfortunately, the non-Gaussian nature of the trait considered does not allow us to derive standardized, and thus comparable, estimates of the additive genetic variance component V_A (because such estimates will always depend on the arbitrary choice of the residual variance V_R, see above). Second, the low heritability estimate may result from methodological difficulties to reliably estimate variance components. Note that we

were interested in a female's inherent propensity for genetic polyandry on the latent-scale. Each individual trait estimate, a female's tendency to give birth to litters sired by multiple fathers, is based on few observations only: the number of litters a female gave birth to. With an average of 3 litters per female, the sampling error is large, potentially making it difficult to derive robust estimates of the behavioural trait of interest. The fact that we detected little evidence for systematic individual differences (measured by V_{PE}), may be a further indication of this inherent sampling problem. Thanks to a high-quality dataset and a state-of-the-art statistical method, we were nevertheless able to derive heritability estimates with relatively narrow confidence bands. It hence seems unlikely that the low heritability estimate can exclusively attributed to insufficient statistical power. Finally, it is possible that there is in fact only little heritable and individual variation for polyandry in this system. As discussed in the main text, we are not the first study that finds polyandry to be largely determined by non-genetic factors such as population density.

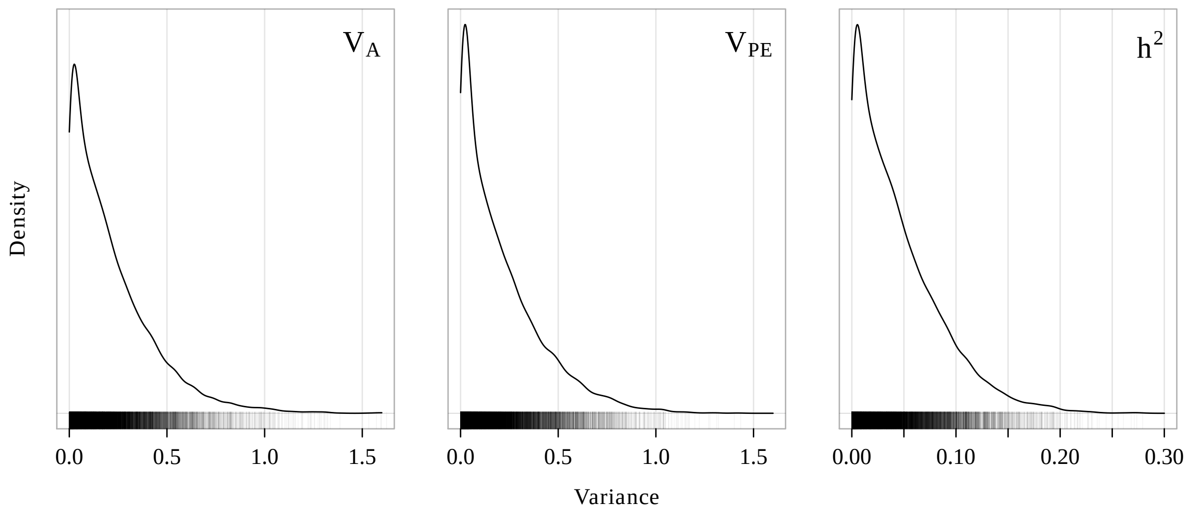

Supplementary Figure 1. The posterior probability density distributions for the two variance components considered in the generalized animal model (additive genetic variance V_A, permanent environmental variance V_{PE} and the resulting density distribution for the heritability h^2.

Supplementary Note 2

Estimating Selection on Polyandry

Modelling Approach We used the total number of offspring at day 13 (time of genetic sampling) produced by a given female during the observation period as a measure of female fitness. We consider this measure a good approximation of lifetime reproduction, although the tenure of some females overlapped with the observation period (right or left-censored). Total offspring number was analysed as a function of multiple mating rate (the focal trait), the t genotype of the female ($+/t$ or $+/+$), the number of reproductive events (litters), and their two-way interactions using a generalized linear model with a Poisson error-distribution and a logarithmic link-function. As a predictor value for an individual female's multiple mating rate, we used the best unbiased linear predictors ($BLUP$) for each individual as derived by the animal model (Supplementary Note 1). We performed a systematic model selection based on $qAIC$ values using the dredge function from the `MuMIn` package in R.

Results Among the 225 females that reproduced at least once, we found considerable overall reproductive skew. In the 4.5 years considered, females produced on average 4.20 litters (ranging up to 13), resulting on average in 13.59 pups that reach the age of 13 days (maximal reproductive success was 56). Neither of the two variables of interest, a female's genetic polyandry frequency ($BLUP$) or t genotype, significantly affected overall reproductive success (Supplementary Figure 2). Accordingly, both variables were removed during model selection (Supplementary Table 1), leaving the number of litters as the sole explanatory variable explaining female fitness (effect size estimate: 0.20, SE = 0.009, $P < 0.001$). Moreover, $+/t$ and $+/+$ females did not differ in the number of litters.

Limitations We find little evidence for selection on genetic polyandry in females. In other words, a female's propensity for polyandry did not affect overall reproductive success in the observed period. The absence of selection may be a statistical artefact. Note that we have used the best linear predictors ($BLUP$) from the animal model to investigate a possible trait-association with reproductive output. Importantly, the animal model included litter size as an explanatory variable (to account for the lower detection probability of genetic polyandry in smaller litters). Hence, the $BLUP$s were already corrected for litter size, arguably one of the main determinants of a female's reproductive success. In light of this, it is probably not surprising that we do not find any effect of polyandry on overall reproduction. Alternatively, we have analysed the effect of average female

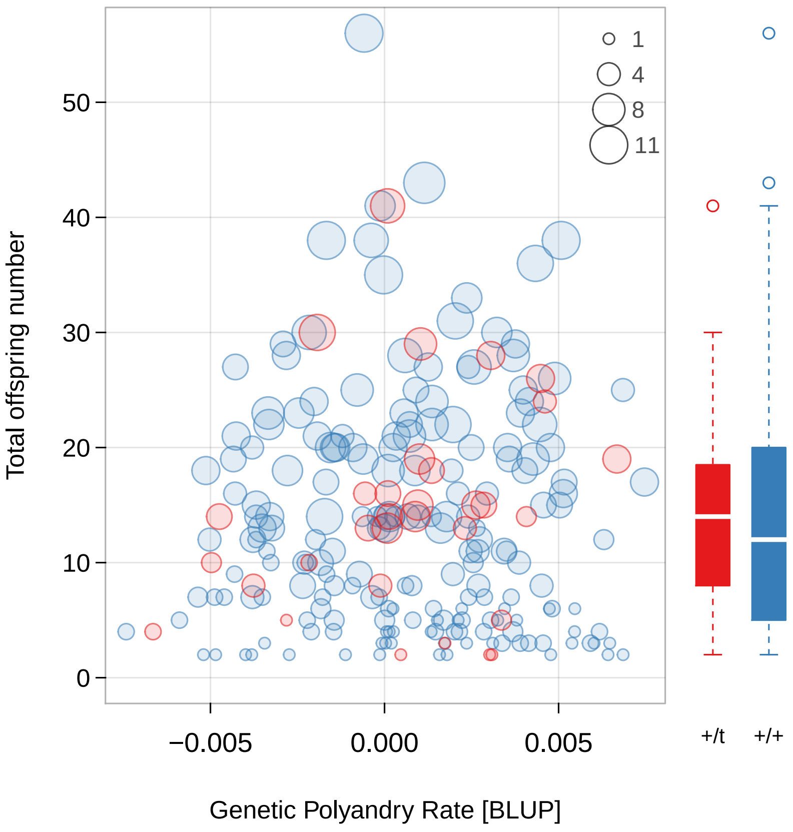

Supplementary Figure 2. Lifetime reproductive success of 225 females as a function of the propensity for polyandry (as calculated by the animal model using BLUPs). An individual's t genotype is represented by red for $+/t$ and blue for $+/+$ individuals. Dot size is proportional to the number of reproductive events (as indicated by the legend in the top-right corners). Boxplots show the difference in reproductive success as a function of t genotype only (showing median (line), 0.25 and 0.75 quartiles (box) $\pm 1.58 *$ inter-quartile range (whiskers)). Source data are provided as a Source Data file.

polyandry rates on reproduction, hence using a predictor variable that is not corrected for litter size. In this case, we find a statistically relevant, positive relationship with reproductive output. However, this analysis has the reverse problem. Hence, it is difficult to assess how much of this positive relationship is biologically relevant, and how much can be attributed to the lower detection probability of genetic polyandry in smaller litters. This is a common problem of studies that analyse the fitness consequences of mating partner number (e.g. Gerlach et al.[2]) and, to our knowledge, there is no straight-forward solution. Note that, irrespective of these methodological difficulties, we expected selection gradients on polyandry to differ between females of different t genotype, as they should affect both genotypes in similar fashion, which we did not detect (see main text).

	(Intercept)	Genotype	Polyandry Rate (BLUP)	Litter Number	G:PR	G:LN	PR:LN	df	logLik	qAIC	Δ
Best Model	1.60		0.20					2	-692.74	683.29	0.00
Model 1	1.63	-0.04	0.20					3	-692.45	685.01	1.72
Model 2	1.59	0.00	0.20	46.93		-52.14		5	-688.50	685.15	1.85
Model 3	1.60		0.20	1.53				3	-692.71	685.26	1.97
Model 4	1.48	0.12	0.22	48.50	-0.02	-53.63		6	-688.07	686.73	3.43
Model 5	1.55	0.05	0.22		-0.02			4	-692.18	686.74	3.45
Model 6	1.59	0.00	0.20	38.66		-52.43	1.69	6	-688.22	686.88	3.58
Model 7	1.63	-0.04	0.20	1.53				4	-692.42	686.98	3.69
Model 8	1.60		0.20	-5.60			1.42	4	-692.51	687.07	3.78
Model 9	1.48	0.13	0.22	38.80	-0.02	-53.93	1.98	7	-687.70	688.36	5.07

Reference level for Genotype: $+/t$

Supplementary Table 1. Model selection and summary statistics of the ten best models (ΔqAIC $<$ 10) analysing female reproductive reproductive success. Female reproductive success was modelled as a function of her t genotype ($+/t$ (ref) and $+/+$), polyandry rate (as estimated by the animal model, BLUP), and the number of litters, and their two-way interactions. Effect sizes are shown on the transformed scale (exponential).

Supplementary Note 3

A Model of Polyandry and Gene Drive

The basic theoretical argument as to how sperm competition will affect t haplotype frequencies in a population has been developed previously. Haig and Bergstrom[5] considered the two most extreme cases, where a female either mates with one male only (monandry) or with the entire male population. Manser et al.[8] examined a model where females either mated with one or two males based on numerical solutions. Here, we develop some analytical solutions with respect to the longterm t expectations (equilibria) and their stability. The model is (partly) based on Manser et al.[8] and Manser et al.[7].

We consider a population of infinite size with non-overlapping generations. For simplicity, we assume that, apart from t/t lethality, there are no survival differences between genotypes (see Manser et al.[9] for an examination of this case). Let y be the frequency of $+/t$ heterozygote adult individuals in the current generation g. Note that, since t/t are not viable, the frequency of $+/+$ homozygotes is simply given by $1 - y$. To calculate the frequency change of $+/t$ individuals Δy from the current (g) to the next, non-overlapping generation ($g + 1$), individuals in the selection lines undergo the following life cycle.

Mating and Fertilisation The key quantity in the model is the probability that a given female is fertilised by a t sperm s, which will be a composite measure of the probability that a female mates with a given male and the probability of t fertilisation given mating. For simplicity, we assume that females randomly mate either once (at probability $1 - \pi$) or twice (at probability π). Parameter π hence measures the polyandry frequency in the population.

The probability of t fertilisation in the case where the female mates with a single male (s_1) will simply be the product of the probability of mating with a $+/t$ male (y) and the probability of t fertilisation (d), $s_1 = yd$. Parameter d here measures the strength of gene drive, where $d = 0.5$ corresponds to Mendelian inheritance and $d = 1$ to complete drive.

In the case where a female mates with two males, there are two ways in which a female can be fertilised by a t sperm (s_2). First, she may mate with two $+/t$ males (at probability y^2), in which case the probability of t fertilisation would be $y^2 d$. Alternatively, she may mate with both a $+/t$ and a $+/+$ male (at probability $2y(1 - y)$). Crucially, we here assume that the $+/t$ males have a sperm competitiveness r relative to $+/+$ males (whose competitiveness equals unity). A $+/t$ male thus only fertilises $\frac{r}{r+1}$ eggs when competing against a wildtype male, of which d will be t. Overall,

we have $s_2 = y^2 d + 2y(1-y)d\frac{r}{r+1}$. If we take sum over both cases $s = (1-\pi)s_1 + \pi s_2$ and simplify, we get

$$s_2 = yd\left(1 - \pi(1-y)\frac{1-r}{1+r}\right). \tag{2}$$

As expected, sperm competition only causes deviations from the monandry case (yd) if females mate with multiple males ($\pi > 0$), drive males differ in their sperm competitive ability ($r \neq 0$), and if wildtype males are present in the population ($1 - y > 0$). The last condition is due to the fact that $+/t$ sperm competition disadvantage is only relevant in matings that involve wildtype males, which will increase with their frequency.

Offspring Production and Embryo Lethality Next, we calculate the proportion of $+/t$ individuals produced in the population in the next, non-overlapping generation $y(g+1)$ (after t/t embryo mortality). Because segregation ratios are Mendelian in females, the fraction of t eggs in the population will simply be

$$e = \frac{y}{2}. \tag{3}$$

Heterozygote $+/t$ zygotes form if a t egg is fertilized by a $+$ sperm (at frequency $e(1-s)$) or if a $+$ egg is fertilized by a t sperm (at frequency $(1-e)s$). $y(g+1)$ will then be the fraction of heterozygotes divided by the total amount of individuals which survive into adulthood. t/t homozygotes, which are formed at a frequency $e\,s$, perish *in utero*, the amount of live offspring is thus $1 - e\,s$. We have the following recurrence relation

$$y(g+1) = \frac{e(1-s) + (1-e)s}{1 - es}. \tag{4}$$

A Monandry

The recurrence equation 4 allows us to derive the equilibrium frequency $\hat{y}$ where the change in t frequency $\Delta y = y(g+1) - y(g) = 0$. We first calculate the equilibria under monandry where $\pi = 0$, thus $s = dy$. If we substitute into equation 4, we recover the three equilibria identified in the classic model by Bruck [1]

$$\hat{y}_1 = 0, \qquad \hat{y}_2 = \frac{d + \sqrt{d(1-d)}}{d}, \qquad \hat{y}_3 = \frac{d - \sqrt{d(1-d)}}{d}, \tag{5}$$

of which only the $\hat{y}_1$ (which is unstable for $d > 0.5$, hence the driver can invade, see below) and $\hat{y}_3$ (which is stable for $d > 0.5$) are biologically feasible (as values for $\hat{y}_2$ are outside $[0,1]$ if $d \in [0,1]$).

B Polyandry

Secondly, we examine a scenario where polyandry and sperm competition occurs ($\pi > 0$). Substituting 2 and 3 into our recurrence equation 4, we get

$$y(g+1) = \frac{2y\Big(y(y-1)(\pi(r-1)+(1-r)\pi - r - 1)d + (1/2)r + 1/2\Big)}{y^2(\pi(r-1)y + (1-r)\pi - r - 1)d + 2r + 2}. \quad (6)$$

Fixed Points Again, we calculate equilibrium points by setting $\Delta y = y(g+1) - y(g) = 0$. For Eq. 6, we get four equilibria, $\hat{y}_1 = 0$ and three relatively complicated expressions (available on demand). For the simpler case where drive is complete ($d = 1$) we again have four equilibria

$$\hat{y}_1 = 0, \qquad \hat{y}_2 = 1,$$
$$\hat{y}_3 = \frac{1 + r + \pi r - 2\pi + \sqrt{1 + r^2 + 2r - 4\pi^2 - 4\pi^2 r^2 + 8\pi^2 r}}{2\pi(r-1)},$$
$$\hat{y}_4 = \frac{1 + r + \pi r - 2\pi - \sqrt{1 + r^2 + 2r - 4\pi^2 - 4\pi^2 r^2 + 8\pi^2 r}}{2\pi(r-1)}. \quad (7)$$

of which $\hat{y}_3$ is outside the $[0, 1]$ boundary and thus not biologically feasible for $0 < r, \pi < 1$.

Invasion Analysis Examining the stability of the internal equilibria is difficult. Instead, let us focus on invasion criteria of the two alleles. The stability of $\hat{y}_1 = 0$ will determine whether the t haplotype can invade a population that is fixed for the wildtype allele. Calculating the first order derivative of Eq. 6 at $\hat{y}_1 = 0$, $\lambda_1 \equiv \frac{dy(n+1)}{dy}\Big|_{y=\hat{y}_1}$ gives

$$\lambda_1 = \frac{1}{2} + (1-\pi)d + \pi d \frac{2r}{1+r}. \quad (8)$$

This expression is quite intuitive to interpret. It is positive for all parameter values ($0 < d < 1$ and $0 < r < \infty$) and thus, the t haplotype can invade if $\lambda_1 > 1$. Drive can thus spread in a population whenever

$$d\left(1 - \pi\frac{1-r}{1+r}\right) > \frac{1}{2} \qquad \text{or} \qquad \pi < \frac{d - \frac{1}{2}}{d} \cdot \frac{1+r}{1-r}. \quad (9)$$

Once again (as in Eq. 2), the term $\pi\frac{1-r}{1+r}$ measures the effect of polyandry on the ability of drive invasion. In the absence of polyandry ($\pi = 0$), the driver can invade as soon as we have gene drive ($d > 0.5$), a result that has been reported previously[1]. With polyandry ($\pi > 0$), it is easy to see how low drive male sperm competitiveness ($r < 1$) and high polyandry rates (π) make the invasion condition more restrictive. For example, if all females in the population mate multiply ($\pi = 1$)

and drive is complete ($d = 1$), 9 reduces to $r > \frac{1}{3}$, thus t male sperm competitiveness has to be greater than 1/3 for the t haplotype to invade. If drive males have the same competitiveness as wildtype males ($r = 1$), $\frac{1-r}{1+r} = 0$, and we again recover the monandry model condition. The right inequality in 9 is rearranged such that we have the polyandry threshold above which the t can no longer establish, which maybe useful for practical purposes. Again, the regime of polyandry rates that impede drive spread gets larger if drive is ineffective and drive sperm disadvantage is small.

We can ask the reverse question and analyse the circumstances under which the wildtype allele can invade a population where all individuals are heterozygotes (at $\hat{y}_2 = 1$). In this case, we have

$$\lambda_2 = \frac{2d^2r - d\pi r + 2d^2 + d\pi - 3dr - 3d + 2r + 2}{(d-2)^2(r+1)}, \tag{10}$$

which, again, is positive for all biologically relevant values. In this case, we find that the wildtype allele can invade a population if $r < -\frac{d^2+d\pi+d-2}{d^2-d\pi+d-2}$.

Supplementary Note 4

Estimating Model Parameters

Analyses performed in this study and Sutter and Lindholm[14] allowed us to obtain reliable estimates of all model parameters for our study population (Supplementary Table 2).

Gene Drive d. The strength of gene drive in our study population during the observation period was $d = 0.912$ [0.787, 0.977]. Note that this estimate is very much in line with previous measures, both from data collected in the population itself[8] as well as from laboratory descendants derived from the study population[6].

Sperm Competitiveness r. Sutter and Lindholm[14] have explicitly measured the sperm competitiveness of $+/t$ relative to $+/+$ males in competitive mating experiments using descendants from the study population. Of 57 competitive matings, $+/t$ males only managed to fertilise 11% of a total of 440 embryos when competing against wildtype males (exact predictions and 95% CI: 11.3% [6.2%,19.6%]). This figure is based on paternity share of the $+/t$ male P_t, which is slightly different to our sperm competitiveness measure here. We have $P_t = \frac{r}{r+1}$ or, equivalently, $r = \frac{P_t}{1-P_t}$, which gives us $r = 0.126$ [0.066, 0.244]. Note that the magnitude of the sperm competitive disadvantage suggests that t haplotypes cause more damage to $+/t$ ejaculates that expected based on the numeric reduction of sperm due to $+$ sperm reduction alone. In the latter scenario, a $+/t$ male's sperm competitiveness would be indirectly proportional to drive strength d as $r[d] = \frac{1}{2d}$[10]. For $d = 0.912$, we would thus expect $+/t$ male sperm competitiveness to be roughly halfed ($r = 0.54$), but actual laboratory estimates are significantly lower than that[10;14].

Polyandry Frequency π. In this study, we have estimated (genetic) polyandry rates π_g based on the paternity information. This is likely to underestimate the actual (behavioural) polyandry rate π in the population, as not all males are necessarily successful in fertilisation ($\pi \geq \pi_g$). However, we can use the polyandry model above to get an approximate idea of the discrepancy between genetic and behavioural polyandry $\Delta\pi = \pi - \pi_g$. Importantly, and additional to the three model parameters (π, r, d) and drive genotype frequency (y), the probability of misassigning a litter $\Delta\pi$ is dependent on the sample size, i.e. the litter size L. For example, in a species where litter size is only one, paternity information is completely uninformative with respect to detecting polyandry.

Inferring Behavioural Polyandry Rates To calculate $\Delta\pi$, we need to determine the frequency of all 8 polyandrous mating combinations $f_{m,n,o}$ as well as the probability of paternity for either of the

males in a particular mating combination $P_{m,n,o}$. Variables n, m, o here denote the genotypes of the female (o) and the two males (m, n) involved in a given polyandrous mating combination (with $m, n, o = 1$ for drive carriers $+/t$ and $m, n, o = 0$ for homozygote wildtypes $+/+$).

The probability of an ordered mating between a female of genotype o and two males of genotype m and n, respectively, under random mating will be

$$f_{m,n,o} = \quad y^k \, (1-y)^{3-k}, \tag{11}$$

where $k = m + n + o$ counts the number of $+/t$ genotypes in the mating and y denotes the frequency of $+/t$ genotypes (also see Supplementary Table 3).

The probability of paternity $P_{m,n}$ of a male of genotype m when competing against a male of genotype n at fertilisation will then be

$$P_{m,n} = \frac{1 - m(1-r)}{2 - (m+n)(1-r)} \tag{12}$$

where r again measures $+/t$ male sperm competitiveness relative to $+/+$ wildtypes. However, things are slightly more complicated because we are not measuring paternity probabilities at fertilisation but at birth. We thus have to account for t/t embryo lethality— every time an embryo of a $+/t$ female is fertilised by a $+/t$ male, a fraction $\frac{d}{2}$ will perish due to t/t lethal effects. Hence, at birth, we have the following paternity probabilities (again summarised in Supplementary Table 3)

$$P'_{m,n,o} = \frac{1 - m(1 - r(1 - \frac{od}{2})}{2 - (m+n)(1 - r(1 - \frac{od}{2}))}. \tag{13}$$

We are now equipped to calculate $\Pr[\Delta\pi|\pi]$ the probability of missing a polyandrous mating

	Definition	Best Estimate [95% CI]	Source
π_g	Frequency of litters with > 1 genetic father	0.473 [0.436, 0.511]	This study
d	Strength of gene drive	0.912 [0.787, 0.977]	This study
r	Sperm competitiveness of $+/t$ males	0.126 [0.066, 0.244]	Sutter and Lindholm [14]
L	Litter size at 13 days of age	4.10 [3.95, 4.25]	This study

Supplementary Table 2. Model parameters, their definitions, and best estimates from the study population as measured in this and previous studies.

given that a polyandrous mating occurs. We miss a multiple mating whenever *all* L offspring of a particular litter are sired by either only the focal male m, which occurs with probability $[P'_{m,n,o}]^L$, *or* his rival n, which occurs at probability $[1 - P'_{m,n,o}]^L$. We now simply sum across all $2^3 = 8$ mating combinations

$$\begin{aligned}\Pr(\Delta\pi|\pi) &= \sum_{m=0}^{1}\sum_{n=0}^{1}\sum_{o=0}^{1} f_{m,n,o}\left(\left[P'_{m,n,o}\right]^L + \left[1 - P'_{m,n,o}\right]^L\right)\\ &= (y^3 + y^2(1-y) + (1-y)^2 y + (1-y)^3)\left(\left[\frac{1}{2}\right]^L + \left[\frac{1}{2}\right]^L\right)\\ &+ 2y^2(1-y)\left(\left[\frac{r(1-\frac{d}{2})}{1+r(1-\frac{d}{2})}\right]^L + \left[\frac{1}{1+r(1-\frac{d}{2})}\right]^L\right)\\ &+ 2y(1-y)^2\left(\left[\frac{r}{1+r}\right]^L + \left[\frac{1}{1+r}\right]^L\right)\end{aligned} \tag{14}$$

The overall difference between behavioural and genetic polyandry will be $\Delta\pi = \pi - \pi_g = \pi \Pr(\Delta\pi|\pi)$. Solving for π, we have

$$\pi = \frac{\pi_g}{1 - \Pr(\Delta\pi|\pi)}. \tag{15}$$

We can now substitute our best estimates for our model parameters (r, d, π, see Supplementary Table 2) into Eq. 15. Average litter size across the entire study period at genetic sampling was $L = 4.10$ [3.95, 4.25]. The average frequency of $+/t$ genotypes throughout the observation period was $y = 0.118$ [0.108, 0.130]. We have $\pi = 0.609$ [0.562, 0.658]. Hence, the proportion of polyandrous litters that are missed based on this simple model is $\Delta\pi = \pi - \pi_g = 13.6\%$ or, equivalently, 22.4% of the polyandrous litters.

Female	Male_1	Male_2	Mating $f_{m,n,o}$	$P'_{m,n,o}$	$1 - P'_{m,n,o}$
$+/t$	$+/t$	$+/t$	y^3	$\frac{1}{2}$	$\frac{1}{2}$
$+/t$	$+/t$	$+/+$	$2y^2(1-y)$	$\frac{r(1-\frac{d}{2})}{1+r(1-\frac{d}{2})}$	$\frac{1}{1+r(1-\frac{d}{2})}$
$+/t$	$+/+$	$+/+$	$y(1-y)^2$	$\frac{1}{2}$	$\frac{1}{2}$
$+/+$	$+/t$	$+/t$	$y^2(1-y)$	$\frac{1}{2}$	$\frac{1}{2}$
$+/+$	$+/t$	$+/+$	$2y(1-y)^2$	$\frac{r}{1+r}$	$\frac{1}{1+r}$
$+/+$	$+/+$	$+/+$	$(1-y)^3$	$\frac{1}{2}$	$\frac{1}{2}$

Supplementary Table 3. Different polyandrous unordered mating combinations, their frequency f, and the expected paternity shares of the two males at birth (after t/t mortality).

Supplementary References

[1] Bruck, D., 1957. Male segregation ratio advantage as a factor in maintaining lethal alleles in wild populations of house mice. Proceedings of the National Academy of Sciences of the United States of America 43:152–158.

[2] Gerlach, N., M. JW, P. Parker, and E. Ketterson, 2012. Reinterpreting bateman gradients: multiple mating and selection in both sexes of a songbird species. Behavioral Ecology 23:1078–1088.

[3] Hadfield, J., 2012. MCMCglmm course notes.

[4] Hadfield, J. D., A. J. Wilson, D. Garant, B. C. Sheldon, and L. E. Kruuk, 2010. The misuse of BLUP in ecology and evolution. The American Naturalist 175:116–125.

[5] Haig, D. and C. Bergstrom, 1995. Multiple mating, sperm competition and meiotic drive. Journal of Evolutionary Biology 8:265–282.

[6] Lindholm, A. K., K. Musolf, A. Weidt, and B. König, 2013. Mate choice for genetic compatibility in the house mouse. Ecology and Evolution 3:1231–1247.

[7] Manser, A., S. J. Cornell, A. Sutter, D. V. Blondel, M. Serr, J. Godwin, and T. A. Price, 2019. Controlling invasive rodents via synthetic gene drive and the role of polyandry. Proceedings of the Royal Society B 286:20190852.

[8] Manser, A., A. K. Lindholm, B. König, and H. C. Bagheri, 2011. Polyandry and the decrease of a selfish genetic element in a wild house mouse population. Evolution 65:2435–2447.

[9] Manser, A., A. K. Lindholm, B. K. o nig, and H. C. Bagheri, 2012. The effect of polyandry on a distorter system with differential viabilities in the sexes. Journal of Communicative and Integrative Biology 5:550–552.

[10] Manser, A., A. K. Lindholm, L. W. Simmons, and R. C. Firman, 2017. Sperm competition suppresses gene drive among experimentally evolving populations of house mice. Molecular Ecology 14:189.

[11] Morrissey, M. B. and A. J. Wilson, 2010. pedantics: an r package for pedigree-based genetic simulation and pedigree manipulation, characterization and viewing. Molecular Ecology Resources 10:711–719.

[12] Nakagawa, S. and H. Schielzeth, 2010. Repeatability for gaussian and non-Gaussian data: a practical guide for biologists. Biological Reviews of the Cambridge Philosophical Society 85:935–956.

[13] Postma, E., F. Heinrich, U. Koller, R. J. Sardell, J. M. Reid, P. Arcese, and L. F. Keller, 2011. Disentangling the effect of genes, the environment and chance on sex ratio variation in a wild bird population. Proceedings of the Royal Society B 278:2996–3002.

[14] Sutter, A. and A. K. Lindholm, 2015. Detrimental effects of an autosomal selfish genetic element on sperm competitiveness in house mice. Proceedings of the Royal Society B 282:20150974.

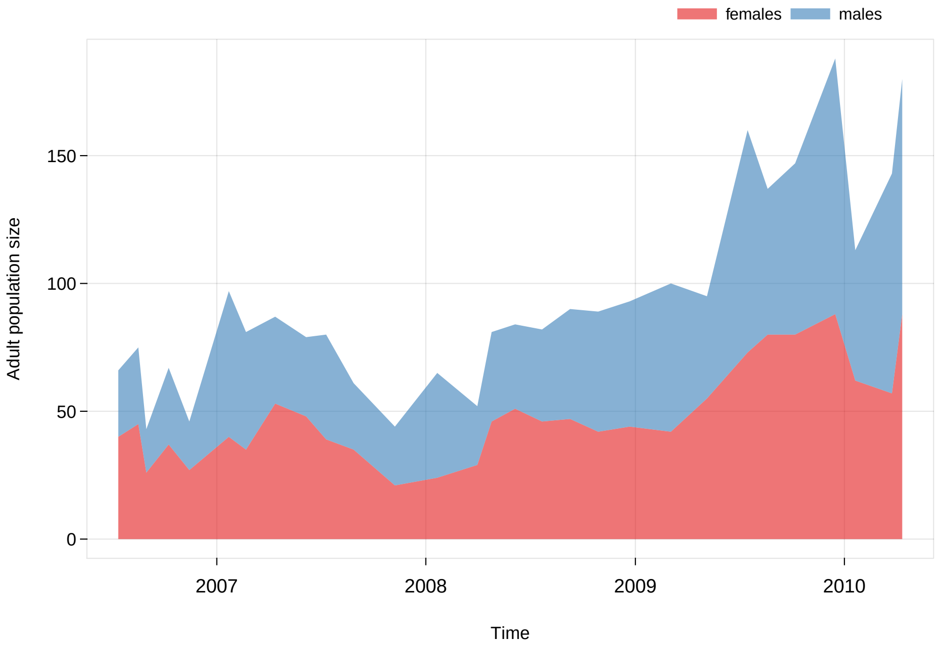

Supplementary Figure 3. Adult male and female population size as registered during population-monitoring events during the observation period. Population density varied considerably, and overall increased in the 4.5 year observation period. Source data are provided as a Source Data file.

	(Intercept)	Genotype	Litter Number	Sperm Comp	G:LN	G:SC	LN:SC	df	logLik	qAIC	Δ
Best Model	3.01	-0.62	0.14	-1.20	-0.05	0.79		6	-928.77	514.75	0.00
Full Model	2.71	-0.47	0.18	-1.02	-0.05	0.71	-0.02	7	-925.07	514.76	0.01
Model 1	1.76	0.54	0.19	-0.35	-0.04		-0.03	6	-933.26	517.17	2.42
Model 2	3.25	-0.88	0.10	-1.06		0.66		5	-938.17	517.82	3.07
Model 3	2.01	0.48	0.14	-0.46	-0.04			5	-938.38	517.94	3.19
Model 4	3.07	-0.82	0.13	-0.95		0.62	-0.02	6	-935.34	518.30	3.55
Model 5	2.46		0.10	-0.46				3	-947.30	518.74	3.99
Model 6	2.32		0.13	-0.38			-0.02	4	-944.26	519.11	4.36
Model 7	2.37	0.10	0.10	-0.46				4	-946.45	520.29	5.53
Model 8	2.23	0.10	0.13	-0.38			-0.02	5	-943.30	520.59	5.84

Reference level for Genotype: $+/t$

Supplementary Table 4. Model selection and summary statistics of the ten best models (Δ qAIC$<$ 10) analysing male reproductive success. Male reproductive success was modelled as a function of t genotype ($+/t$ (ref) and $+/+$), the level of sperm competition (average number of rival males per reproductive event), the number of litters a male contributed to, and their two-way interactions. Effect sizes are shown on the transformed scale (exponential).
