# Supplementary material for: Polyandry blocks gene drive in a wild house mouse population
Source: Nat Commun. 2020 Nov 4;11:5590. doi: 10.1038/s41467-020-18967-8 (PMC7643059; doi:10.1038/s41467-020-18967-8)
Supplement: Supplementary file 3 — Reporting Summary [file 41467_2020_18967_MOESM3_ESM.pdf]

nature research

Corresponding author(s): Andri Manser

Last updated by author(s): 2020/09/08

Reporting Summary

Nature Research wishes to improve the reproducibility of the work that we publish. This form provides structure for consistency and transparency in reporting. For further information on Nature Research policies, see our Editorial Policies and the Editorial Policy Checklist.

Statistics

For all statistical analyses, confirm that the following items are present in the figure legend, table legend, main text, or Methods section.

n/a	Confirmed	
☐	☒	The exact sample size (*n*) for each experimental group/condition, given as a discrete number and unit of measurement
☐	☒	A statement on whether measurements were taken from distinct samples or whether the same sample was measured repeatedly
☐	☒	The statistical test(s) used AND whether they are one- or two-sided<br>*Only common tests should be described solely by name; describe more complex techniques in the Methods section.*
☐	☒	A description of all covariates tested
☐	☒	A description of any assumptions or corrections, such as tests of normality and adjustment for multiple comparisons
☐	☒	A full description of the statistical parameters including central tendency (e.g. means) or other basic estimates (e.g. regression coefficient) AND variation (e.g. standard deviation) or associated estimates of uncertainty (e.g. confidence intervals)
☐	☒	For null hypothesis testing, the test statistic (e.g. *F*, *t*, *r*) with confidence intervals, effect sizes, degrees of freedom and *P* value noted<br>*Give P values as exact values whenever suitable.*
☐	☒	For Bayesian analysis, information on the choice of priors and Markov chain Monte Carlo settings
☒	☐	For hierarchical and complex designs, identification of the appropriate level for tests and full reporting of outcomes
☒	☐	Estimates of effect sizes (e.g. Cohen's *d*, Pearson's *r*), indicating how they were calculated

Our web collection on statistics for biologists contains articles on many of the points above.

Software and code

Policy information about availability of computer code

Data collection	R (Version 3.5.2)
Data analysis	R (Version 3.5.2): R-packages: MCMCglmm (v2.29), Pedantics (v1.7), MuMIn (v1.43.15), lme4 (v1.1-21), and RColorBrewer (v1.1-2); Maple Software 2016; Cervus 3.0

For manuscripts utilizing custom algorithms or software that are central to the research but not yet described in published literature, software must be made available to editors and reviewers. We strongly encourage code deposition in a community repository (e.g. GitHub). See the Nature Research guidelines for submitting code & software for further information.

Data

Policy information about availability of data

All manuscripts must include a data availability statement. This statement should provide the following information, where applicable:

- Accession codes, unique identifiers, or web links for publicly available datasets
- A list of figures that have associated raw data
- A description of any restrictions on data availability

The data used in this study is available on Figshare (https://doi.org/10.6084/m9.figshare.12706595.v1). Source data are provided with this paper.

Field-specific reporting

Please select the one below that is the best fit for your research. If you are not sure, read the appropriate sections before making your selection.

☐ Life sciences ☐ Behavioural & social sciences ☒ Ecological, evolutionary & environmental sciences

For a reference copy of the document with all sections, see nature.com/documents/nr-reporting-summary-flat.pdf

Ecological, evolutionary & environmental sciences study design

All studies must disclose on these points even when the disclosure is negative.

Study description	The study makes use of a long-term data set that has been collected on free-living population of house mice. It is based on field observations that were not experimentally manipulated. For further details on the methods, please refer to the following manuscripts.<br>1) König & Lindholm (2012), "The complex social environment of female house mice (Mus domesticus)." Cambridge Studies in Morphology and Molecules: New Paradigms in Evolutionary Bio, 114-134.<br>2) König et al. (2015), "A system for automatic recording of social behavior in a free-living wild house mouse population." Animal Biotelemetry, 39.
Research sample	For the purpose of this study, we attempted to sample *all* house mice (Mus musculus domesticus) that were born in our study site (barn outside Zürich, Switzerland) during the 4.5 year period between January 2006 and June 2010. We ended up with a sample of 3,127 pups born in 1,015 litters from 279 females and 249 males. Our sample thus comprised of both sexes and all age classes (from newborn to adult, maximum life span around 3 years). The study period was chosen such that the gene of interest (t haplotype) was still present at sufficiently high frequency to study its impact. (Re-)capturing of mice at subadult and adult stage suggest that our sampling method finds more than 95% of pups born in the population, we thus think our sample is representative of the study population as whole.
Sampling strategy	Because the study is based on field observation, the sample size was the result of the number of litters that occurred naturally during the observation period. Sample size was thus not predetermined by the researchers.
Data collection	Nest boxes were checked for newly born litters on a weekly basis. About every 7 weeks, the entire population was captured, sexed, and individually marked (using RFID tags; Trovan ID-100A implantable microtransponder: weight: 0.1 g, length: 11.5 mm; diameter: 2.1 mm), allowing us to estimate the overall density in the population. Ear punches (Easy-grip Combo Ear Punch, 1mm) were taken from 13 day old pups and from sexually mature adults for genotype diagnosis and parentage analysis. Data was collected and recorded by students and staff of our research group over the years.
Timing and spatial scale	January 2006 to June 2010 in a barn building outside Zürich, Switzerland (dimensions: 72 m^2, coordinates: 47.418891 N, 8.726893 E, altitude: 556 m.a.s.l.). For frequency of data collection, see just above.
Data exclusions	Offspring were excluded if parentage could not be assigned at a 95% level of confidence. This criterion was established prior to parentage analysis.
Reproducibility	The project is based on observational and not experimental data. The longterm field study as a whole represents a very large, 18 year research effort that would be very difficult to reproduce / replicate.
Randomization	The project is based on a longterm field observations (18 years) without experimental manipulation. There were thus no experimental/treatment groups. t genotype of animals, the main covariate in our statistical analysis, is a property of the animal that cannot be assigned by the researcher. All individuals that were caught and sampled during the observation period were used in our analysis.
Blinding	The data were collected blind, as there is no external phenotype associated with the genotype which was the main focus of the study, and genotype was only diagnosed after all the data were collected.

Did the study involve field work? ☒ Yes ☐ No

Field work, collection and transport

Field conditions	The population lives indoors (as is usual for house mice). The temperature range is approximately between -10 and 30 degree Celcius.
Location	The study population lives in a barn outside Illnau (Switzerland). Coordinates: 47.418891 N, 8.726893 E; Altitude: 556 m.a.s.l.
Access & import/export	Sampling and data collection were approved by the Swiss animal welfare body (Veterinary Office of Canton Zürich). Permit numbers: 210/2003, 215/06, 51/10.
Disturbance	We tried to minimize disturbance as much as possible. However, regular checks of nests for young as well as occasional capture of the entire population (see above) was necessary to collect the information required for this study.

Reporting for specific materials, systems and methods

We require information from authors about some types of materials, experimental systems and methods used in many studies. Here, indicate whether each material, system or method listed is relevant to your study. If you are not sure if a list item applies to your research, read the appropriate section before selecting a response.

Materials & experimental systems

n/a	Involved in the study
[x]	[ ] Antibodies
[x]	[ ] Eukaryotic cell lines
[x]	[ ] Palaeontology and archaeology
[ ]	[x] Animals and other organisms
[x]	[ ] Human research participants
[x]	[ ] Clinical data
[x]	[ ] Dual use research of concern

Methods

n/a	Involved in the study
[x]	[ ] ChIP-seq
[x]	[ ] Flow cytometry
[x]	[ ] MRI-based neuroimaging

Animals and other organisms

Policy information about studies involving animals; ARRIVE guidelines recommended for reporting animal research

Laboratory animals	No laboratory animals were used in this study
Wild animals	This study reports on free-living, wild Mus musculus domesticus of both sexes and all age classes. Nest boxes are checked for newly born litters on a weekly basis. About every 7 weeks, the entire population is captured, sexed, and individually marked, allowing us to estimate the overall density in the population. Ear punches were taken from 13 day old pups and from sexually mature adults for genotype diagnosis and parentage analysis. The study is based on an ongoing, longterm field project of a free-living mouse population and animals were returned to the population after handling. That is, no animals killed either at sampling or the end of the study.
Field-collected samples	No live animals were sampled from the field.
Ethics oversight	Gesundheitsdirektion Veterinäramt of the Canton Zürich (Switzerland). Permit numbers: 210/2003, 215/06, 51/10.

Note that full information on the approval of the study protocol must also be provided in the manuscript.
